# A probable right atrial myxoma prolapsing through the tricuspid valve into the right ventricle: a case report

**DOI:** 10.1186/1757-1626-1-386

**Published:** 2008-12-11

**Authors:** Dike B Ojji, Stella S Ajiduku, Omonuyi O Omonua, Lukman L Abdulkareem, Will Parsonage

**Affiliations:** 1 Cardiology Unit, Department of Medicine, University of Abuja Teaching Hospital, Gwagwalada, Abuja, Nigeria; 2 Department of Cardiology, Royal Brisbane and Women's Hospital, Brisbane, Queensland, Australia

## Abstract

**Introduction:**

Myxoma is the most common primary cardiac tumour. This case report illustrates the case of a probable right atrial myxoma prolapsing through the tricuspid valve into the right ventricle, and the fatal outcome if such a mass is not promptly detected and excised.

**Case presentation:**

A 51-year old man presented with a 1-year history of recurrent pedal and abdominal swelling, and 6-month history of progressive dyspnoea on exertion. Transthoracic echocardiography showed a large right atrial mass prolapsing through the tricuspid valve into the right ventricle. Patient discharged himself against medical advice and died about one hour after getting home while trying to stand up from the sitting position.

**Conclusion:**

Cardiac myxomas should always be considered when the cause of heart failure is not obvious. Transthoracic echocardiography remains an invaluable tool in the diagnosis, and prompt treatment is necessary to avoid fatal outcomes.

## Introduction

Primary tumours of the heart are not common and have been found in only 0.0017% to 0.19% of unselected patients at post mortem[1,2]. The commonest of these primary tumours are myxomas, and of the myxomas 75% occur in the left atrium, and 25% in the right atrium, and occasionally in the ventricle[3].

This case report demonstrates a rare form of a probable cardiac myxoma arising from the right atrium and prolapsing through the tricuspid valve into the right ventricle.

## Case presentation

A 51 year-old Nigerian African male presented December 2007 with a 1-year history of recurrent pedal and abdominal swelling (he had been treated with diuretics intermittently by his General Practitioner). There was also a 6-month history of progressive dyspnoea on exertion with associated orthopnoea and paroxysmal nocturnal dyspnoea. There was no history of chest pain, palpitations nor syncope. He also had fever, progressive weight loss and malaise which started with onset of illness. The patient was not a known hypertensive, diabetic nor asthmatic. There was no history of alcohol ingestion, neither was there any history of cigarette smoking.

Physical examination revealed pitting pedal oedema up to the knees, hepatomegaly of 3 cm below the right costal margin with a liver span of 15 cm and moderate ascites. On cardiovascular system examination, the blood pressure was 100/80 mmHg, the apex beat was diffuse and first and second heart sounds which were distant were heard.

The haemoglobin was 13.8 gm/dl and the white blood cell count was 6.0 × 10^9^/L with lymphocyte count of 55% and neutrophil count of 42%. Total and direct bilirubin levels were elevated at 70.3 μmol/L and 48.1 μmol/L (normal: ≤ 17.1 μmol/L and ≤ 4.3 μmol/L respectively). Aspartate Transaminase, Alanine Transaminase, Alkaline Phosphatase were all elevated at 289 iu/L, 93 iu/L and 68 iu/L respectively (normal being:<37 iu/L, <40 iu/L and 9–35 iu/L respectively). Hepatitis B surface antigen was not detectable while antibody against Hepatitis C virus was detected. An abdominal ultrasound scan showed that the liver was mildly enlarged with a coarse echo pattern.

**Figure 1:**
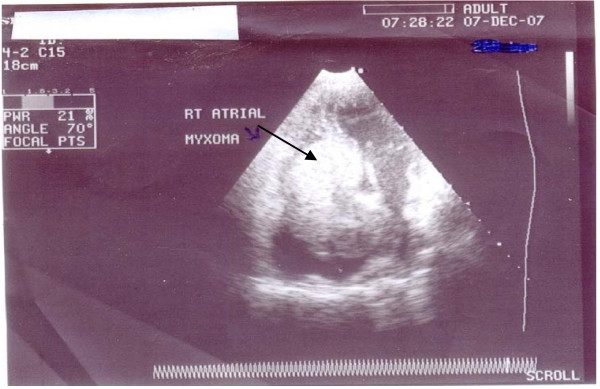
**Four-Chamber View of Patient's Transthoracic Echocardiographic Study**. This figure shows a large echo dense mass arising from the inter-atrial septum within the right atrium, and prolapsing through the tricuspid valve into the right ventricle. The mass measured 5.3 cm by 4.2 cm.

Transthoracic Echocardiography (Fig. 1) showed a large echo-dense mass in the right atrium prolapsing through the tricuspid valve into the right ventricle. It measured 5.3 cm by 4.2 cm.

Our patient discharged himself against medical advice when the nature of illness and treatment modalities were explained to him. Unfortunately, he died about one hour after getting home from the hospital while trying to stand up from the sitting position. No post mortem examination was performed.

## Discussion

Right atrial myxoma accounts for only about 25% of all myxomas [3]. It usually arises from the inter-atrial septum as in the case of our patient. In this patient, the atrial mass was found to prolapse through the tricuspid valve into the right ventricle. A similar right atrial myxoma prolapsing through the tricuspid valve into the right atrium was reported by Soma Guhathakurta and John Riordan [4]. The reason for such prolapse is the attachment of the myxoma on a long stalk.

Myxomas can present at any age group but occurs more often between the 3^rd ^and 6^th ^decades of life [5] as is the case with our patient who was 51 years.

The most common symptoms of right atrial myxoma have been reported to be those of congestive heart failure, while other symptoms vary from constitutional to thrombo-embolic and obstructive [6]. Our patient presented with features of congestive heart failure and constitutional symptoms of fever, malaise and weight loss. Constitutional symptoms in patients with cardiac myxomas have been attributed to cardiac myxomas releasing the cytokin, interleukin-6 which causes inflammatory and auto-immune manifestations [7].

The deranged liver enzymes can be partly explained by hepatic congestion from congestive heart failure.

Our patient died about one hour after getting home from the hospital while trying to get up from the sitting position. We think he suddenly had a complete obstruction of the tricuspid valve which resulted in syncope and sudden death. This phenomenon has been reported as one of the commonest causes of death in right atrial myxoma especially when it extends to the tricuspid valve [8].

## Conclusion

Cardiac myxomas should always be considered when patients present with symptoms of heart failure with uncertain aetiology. In addition, transthoracic echocardiography remains an invaluable tool in the diagnosis of this uncommon condition, while prompt diagnosis and treatment is necessary to avoid fatal outcome.

## Consent

Written informed consent was obtained from the patient's younger brother who is the next of kin for publication of this case report and accompanying images.

## Competing interests

The authors declare that they have no competing interests.

## Authors' contributions

DO was involved in collecting patient details, reviewing the literature and drafted the manuscript as the main author. WP was involved in proof reading the manuscript. SA, OO and LA managed the patient on initial presentation to the hospital. All authors have read and approved the final manuscript.
